# Mitochondrial DNA haplogroups and circulating cell-free mitochondrial DNA as biomarkers of bronchopulmonary dysplasia

**DOI:** 10.1038/s41390-025-04052-7

**Published:** 2025-04-17

**Authors:** Sara María Fernandez-Gonzalez, Andrea Sucasas-Alonso, Vanesa Balboa-Barreiro, Ignacio Rego-Perez, Alejandro Avila-Alvarez

**Affiliations:** 1 Neonatology Department, Complexo Hospitalario Universitario A Coruña (CHUAC), A Coruña, Spain; 2 Biostatistics and Epidemiology Unit, Instituto de Investigación Biomédica de A Coruña (INIBIC), Complexo Hospitalario Universitario de A Coruña (CHUAC), Sergas, Universidade da Coruña (UDC), A Coruña, Spain; 3 Grupo de Investigación en Reumatología, Unidad de Genómica, Instituto de Investigación Biomédica de A Coruña (INIBIC), Complexo Hospitalario Universitario, de A Coruña (CHUAC), Sergas, Universidade da Coruña (UDC), A Coruña, Spain; 4 Grupo de Investigación en Pediatría. Instituto de Investigación Biomédica A Coruña (INIBIC), Complexo Hospitalario, Universitario A Coruña (CHUAC), Sergas, Universidade da Coruña (UDC), A Coruña, Spain

## Abstract

**Background:**

Recognizing which premature infants are at higher risk of developing BPD/death is a challenge in neonatology. The aims of our study are to identify mitochondrial haplogroups and quantify circulating cell-free mitochondrial DNA (ccf-mtDNA) levels in very preterm infants at risk of bronchopulmonary dysplasia (BPD) or death and explore the relationship between these variables and the development of BPD/death.

**Methods:**

Single-center prospective cohort study including preterm infants of ≤32 weeks gestational age (GA) and birth weight ≤1500 g. Clinical variables, mitochondrial haplogroups, and ccf-mtDNA levels were determined. Subsequently, diagnosis and staging of BPD/death were performed, and groups were compared.

**Results:**

The population consisted of 107 newborns (mean GA 28.73 ± 2 weeks; mean birth weight 1,121 ± 332 g). A total of 44 patients (41.1%) presented the outcome of BPD/death without differences in haplogroup distribution and ccf-mtDNA levels between those who survived without BPD (controls). Variables independently associated with BPD/death included GA (*p* < 0.001; OR = 0.36 [95%CI 0.23–0.5]), birth weight (*p* < 0.001; OR = 0.99 [95%CI 0.99–0.99]), maximum FiO_2_ in the delivery room (*p* = 0.001; OR = 1.07 [95%CI 1.03–1.12]), hours on mechanical ventilation (*p* = 0.02; OR 1.02 [95%CI 1.00–1.02]), and postnatal corticosteroids (p < 0.001; OR = 47.12 [95%CI = 5.98–371.1]).

**Conclusion:**

This is the first study to characterize mtDNA haplogroups and ccf-mtDNA in very preterm infants at risk of BPD/death. None of the mitochondrial variables studied were associated with BPD/death. Further research is needed to elucidate the role of mtDNA in BPD.

**Impact statement:**

- Despite advances in perinatal care, bronchopulmonary dysplasia continues to be the most common chronic pulmonary morbidity associated with prematurity.
- Prediction of BPD in early stages is crucial to improve BPD rates, but this remains a major challenge in neonatal units.
- Given that mitochondria play an important role in the inflammatory and oxidative stress responses, we aimed to explore the relationship between mitochondrial haplogroups, circulating cell-free mitochondrial DNA levels, and BPD.
- This is the first work carried out in very preterm infants where mitochondrial haplogroups and the levels ccf-mtDNA are investigated with the intention of discovering a new biomarker for BPD.

## Introduction

Bronchopulmonary dysplasia (BPD) remains a common respiratory sequela of prematurity.^1^ BPD is associated with increased morbidity and mortality,^1^ reduced quality of life, and increased costs and use of healthcare resources.^2^ Despite advances in perinatal management over the past three decades, BPD rates among very preterm infants remain elevated, ranging from 15–50%.^3–5^

It is widely acknowledged that the primary contributors to BPD development include immaturity itself, invasive mechanical ventilation (IMV), and supplementary oxygen.^1^ Over time, this understanding has evolved into a more complex model. It now considers how disruptions in lung development interact with inflammation from different sources, such as perinatal infections, oxidative stress (OS), injury caused by mechanical ventilation, surfactant deficiency, and potential genetic factors yet to be identified.^3^

Some studies have implicated mitochondrial dysfunction in prematurity-related comorbidities^6^, such as BPD. Moreover, mitochondrial dysfunction, characterized by increased free radicals, mtDNA mutations, mtDNA damage, and decreased adenosine triphosphate production, has been linked to lung injury induced by hyperoxia or invasive mechanical ventilation. These findings highlight the importance of studying mitochondria in patients at risk of BPD.

Mitochondria play an important role in the molecular and cellular process involved in inflammatory and OS responses in BPD. Indeed, the mitochondrial respiratory chain is a powerful source of reactive oxygen species, which could be considered a pathogenic agent of many diseases, including BPD.^7^

Mitochondria have their own DNA, and each one contains 5–10 copies of mitochondrial DNA (mtDNA). mtDNA is more vulnerable to oxidative damage than nuclear DNA^8^ due to its lack of histone protection and its less-efficient repair mechanisms.^9^ Certain polymorphisms in coding regions of mtDNA, known as mitochondrial haplogroups, are inherited together. Specific haplogroups can alter mitochondrial activity, promoting the production of free radicals and mtDNA damage,^8^ thereby increasing individual susceptibility to certain pathologies. Recent research has revealed that specific molecular processes begin very early during fetal development or at birth and that preterm infants have a distinct, genetically determined sensitivity to OS.^10^ Few studies have been conducted on preterm infants. However, some evidence suggests that haplogroups L2a and L3 may increase the risk of preterm birth.^11^ Understanding how these haplogroups contribute to disease susceptibility could help identify vulnerable infants early on, particularly in the context of BPD.

Normally found in the mitochondrial matrix,^12^ mtDNA can be released into the cytosol as a result of mitochondrial rupture, a process that results in circulating cell-free mitochondrial DNA (ccf-mtDNA). This release of mtDNA into the extracellular space is not simply a byproduct of cellular injury but acts as a danger-associated molecular pattern (DAMP),^13,14^ which plays a pivotal role in activating the inflammatory response. Elevated levels of ccf- mtDNA have been observed in patients with sepsis,^15^ and severe trauma,^16^ and have been significantly associated with increased mortality risk in patients admitted to intensive care units.^17^ Faust et al. ^18^ found that elevated levels of ccf-mtDNA in patients with sepsis or trauma have been linked to an increased risk of developing acute respiratory distress syndrome within the first 48 hours following the insult. This association suggests that ccf-mtDNA not only serves as a marker of cellular injury but also plays a role in the inflammatory pathways that contribute to the progression of respiratory failure. Given this, we believe that measuring circulating mtDNA levels during the early days of life in preterm infants could provide valuable predictive information. Specifically, we hope to determine whether elevated mtDNA levels could help predict which infants are at greater risk of developing BPD, enabling earlier interventions to either prevent or mitigate the course of the disease.

Hence, the aim of this study was to explore if the levels of ccf-mtDNA at birth or the distribution of mitochondrial haplogroups was different among patients with BPD or death. Our main hypothesis was that certain mitochondrial haplogroups may modify baseline predisposition to the development of BPD in this population. A better understanding of the role of mtDNA in BPD could facilitate the identification of new biomarkers and help improve the prediction of prognosis in early disease stages.

## Materials and methods

This was a prospective cohort study carried out in a tertiary neonatal intensive care unit (NICU). We collected data from preterm infants of gestational age ≤32 weeks and body weight ≤1500 g who were admitted to the NICU between November 2019 and the first week of March 2024. Patients with life-threatening malformations or chromosomal anomalies were excluded. From the included patients, 1 ml of blood was collected within the first 24 hours of life to determine mitochondrial haplogroup and ccf-mtDNA levels. The main study outcome was the combined variable BPD or death by any cause. Therefore, patients with BPD/death constituted the cases group, and survivors without BPD constituted the control group. Patients born to non-Caucasian mothers were excluded from the study for the comparison of haplogroups between cases and controls. The study was approved by the local Clinical Research Ethics Committee (This study was approved by the local Clinical Research Ethics Committee (RESEARCH ETHICS COMMITTEE OF A CORUÑA - FERROL, registration code 2021/349), and for all participants, written parental consent was obtained before inclusion.

### Variables and definitions

Secondary outcomes included BPD, moderate-to-severe BPD, IMV at the 7th day of life, fraction of inspired oxygen (FiO_2_) at the 7th day of life, postnatal corticosteroids, duration of MV and duration of supplemental oxygen. BPD was defined as the need for supplementary oxygen for at least 28 days and classified as moderate or severe depending on oxygen requirements and ventilator support at 36 weeks postmenstrual age (moderate if supplementary oxygen only; severe if respiratory support required).^19,20^ The following variables were recorded and compared between groups: demographic data at birth and at screening (GA, birth weight, sex, Apgar score at 1 and 5 min, temperature at admission); perinatal characteristics (antenatal steroids, maternal age, maternal hypertension, chorioamnionitis, in vitro fertilization, mode of delivery); respiratory support (IMV, non-invasive positive pressure ventilation), surfactant, maximum FiO_2_ in the delivery room, and other relevant clinical neonatal outcomes (necrotizing enterocolitis, patent ductus arteriosus, intraventricular hemorrhage, retinopathy of prematurity, isolated intestinal perforation, mortality, NICU and hospital stay). Only enterocolitis ≥ grade 2, as defined by Bell et al.^21^, was considered. Patent ductus arteriosus was diagnosed by cardiac ultrasound and managed according to local protocols. Intraventricular hemorrhage was defined and graded according to Volpe.^22^ All infants were screened for retinopathy according to national guidelines.^23^ Small for gestational age was defined as a birth weight z-score < −1.5 according to the 2013 Fenton growth charts.

### Genotyping of mtDNA haplogroups

DNA was automatically isolated in a Qiacube instrument (Qiagen) using QIAamp DNA Blood Mini Kit (Qiagen) following the manufacturer´s recommendations. Once isolated, mtDNA haplogroups were assigned in 107 samples following a previously described assay.^24^ Briefly, a multiplex polymerase chain reaction (PCR) was performed to amplify 6 mtDNA fragments containing the informative SNPs that characterize the most common Caucasian mtDNA haplogroups, as well as less common SNPs pooled into the group named “Others”. Next, the resulting PCR fragments were purified and analyzed by single base extension (SBE) assay to further visualize the informative SNPs after loading the SBE product into a SeqStudio genetic analyzer (ThermoFisher Scientific).

### Quantification of circulating cell-free mtDNA

Cell-free mtDNA was isolated from plasma using the MagMax cell-free DNA isolation kit (Applied Biosystems). Briefly, 500-µl plasma samples were added to 500 µl of phosphate-buffered saline (PBS) and then centrifuged at 16,000 *g* for 10 minutes at 4 °C. Supernatants (~1 mL) were retained for the subsequent procedures according to the manufacturer´s recommendations. At the last step, 25 µl of elution buffer was added to resolve the cell-free plasma mtDNA.

Circulating cell-free mtDNA plasma levels were measured by real-time PCR assay, using SYBR green, in a LightCycler 480 system and quantified using the following formula from a previously described assay^25^:

$${{{\rm{c}}}}={{{\rm{Q}}}}\times {{{{\rm{V}}}}}_{{{{\rm{DNA}}}}}/{{{{\rm{V}}}}}_{{{{\rm{PCR}}}}}\times 1/{{{{\rm{V}}}}}_{{{{\rm{ext}}}}}$$

where **c** is the concentration of plasma cell-free mtDNA (copies/µl); **Q** is the quantity of DNA measured by real-time PCR; **V**_**ADN**_ means the total volume of plasma cell-free mtDNA obtained from the extraction (25 µl); **V**_**PCR**_ is the volume of plasma DNA for real-time PCR (5 µl); and V_ext_ is the volume of plasma used for the extraction (500 µl).

### Statistical analyses

A sample size of 100 patients was previously calculated to be able to detect a statistically significant difference of 15% (from 30% to 45%) in the incidence of BPD/death between patients with a given haplogroup and the rest, with a confidence level (1–α) of 95% and a power of 75%.

Descriptive data were presented as mean ± standard deviation (SD) for normally distributed variables, as the median (interquartile range, IQR) for non-normal, and as *n* (%) for qualitative variables. Initially, a univariate analysis was conducted to assess the association between qualitative variables in both groups, primarily the distribution of mitochondrial haplogroups, which were analyzed using the χ^2^ or Fisher’s exact test, as appropriate. Mean comparisons, particularly for ccf-mtDNA levels, were performed using the Student’s *t*-test or the non-parametric median test in cases of non-normally distributed data. Mean comparisons of more than two groups were performed using analysis of variance or, in cases of non-normally distributed data, the non-parametric median test. Correlations between quantitative variables were examined using Pearson’s correlation coefficient or the non-parametric Spearman correlation, if applicable. Logistic regression models were then performed for multivariate analysis, adjusting by GA. For all hypothesis tests, the null hypothesis was rejected with a type I error or alpha level less than 0.05. Analyses were performed using IBM SPSS statistical software for Windows v. 24.0.

## Results

**Fig. 1:**
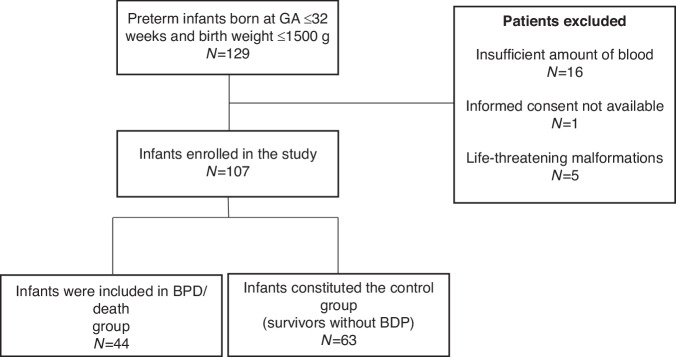
Flowchart depicting the study population. GA gestational age, BPD bronchopulmonary dysplasia.

From November 2019 to March 2024, a total of 129 infants of GA ≤ 32 and birth weight ≤1500 g were admitted to the study unit. After applying exclusion criteria (see flow chart, Fig. 1), a total of 107 newborns (48.6% girls) were finally included in the study. The mean GA and birth weight of the entire cohort were 28.73 ± 2 weeks and 1121 ± 332 g, respectively. A total of 44 patients (41.1%) presented with the combined outcome of BPD/death (cases). Among these, 34 developed BPD, and 10 patients died before BPD could be diagnosed (i.e. before 28 days of life).

### Perinatal characteristics and neonatal outcomes

Some demographic and clinical features differed significantly between patients with BPD/death and those who survived without BPD (see Table 1). Patients with the combined variable BPD/death were younger (27.18 ± 1.8 weeks GA vs. 29.8 ± 1.32 weeks GA; *p* < 0.001), with a lower BW (895 ± 276 g vs. 1278 ± 273 g; *p* < 0.001) and required higher FiO_2_ during DR stabilization (42.4 ± 18.3 vs. 30.8 ± 10.6; *p* 0.001). Moreover, these patients had a longer NICU stay (40.5 ± 22.4 vs. 23.3 vs. 12.7; *p* < 0.001), a longer total hospital stay (80.5 ± 113.9 vs. 51.5 ± 14.6; *p* 0.02), and a higher mortality rate (22.7% vs. 1.5%; *p* < 0.001). Patients with BPD/death also showed a higher incidence of other comorbidities such as severe intraventricular hemorrhage, retinopathy, and enterocolitis and had higher rates of medically-treated patent ductus arteriosus.

**Table 1:** Descriptive and univariate analyses of main perinatal variables and outcomes.

Perinatal characteristics $\bar{X}$± SD (IQR)	Total *N* = 107	BPD/death *N* = 44	No BPD/death *N* = 63	*p*-value	OR	aOR^a^
**Gestational age (weeks)**	**28.73** ± **2.00 (27.2–30.2)**	**27.18** ± **1.8 (26.3–28.2)**	**29.8** ± **1.32 (29–31.1)**	**<0.001**	**0.36 (0.23–0.50)**	**0.4 (0.24–0.67)**
**Birth weight (g)**	**1121** ± **332 (885–1400)**	**895** ± **276 (725–1025)**	**1278** ± **273 (1050–1490)**	**<0.001**	**0.99 (0.99–0.99)**	**0.99 (0.99–1.00)**
*N* (%)						
**SGA**	**17 (15.9)**	**12 (27.9)**	**5 (7.9)**	**0.03**	**3.97 (1.22–15.4)**	**11.24 (2.57–61.18)**
ANS	97 **(**90.7)	39 **(**88.6)	59 **(**93.7)	0.35	0.52 **(**0.12–2.06)	1.16 **(**0.20–6.84)
Maternal hypertension	23 **(**21.5)	10 **(**23.3)	13 **(**21)	0.78	1.14 **(**0.44–2.9)	1.16 **(**0.33–3.96)
Multiple birth	25 **(**23.4)	9 **(**20.5)	16 **(**25.4)	0.55	0.76 **(**0.29–1.88)	0.85 **(**0.26–2.68)
IVF	14 **(**13.1)	5 **(**11.4)	9 **(**14.3)	0.66	0.77 **(**0.22–2.41)	0.27 **(**0.04–1.38)
Female	52 **(**48.6)	20 **(**45.5)	32 **(**50.8)	0.59	0.81 **(**0.37–1.75)	0.41 **(**0.13–1.19)
Chorioamnionitis	21 **(**19.6)	9 **(**20.9)	12 **(**19.7)	0.88	1.08 **(**0.4–2.84)	0.51 **(**0.11–2.00)
Cesarean section	59 **(**55.1)	22 **(**50)	37 **(**58.7)	0.37	0.70 **(**0.32–1.53)	1.30 **(**0.45–4.01)
**Respiratory outcomes** ${\bar{{{\boldsymbol{X}}}}}$± **SD (IQR)**						
**Maximum FiO**_**2**_ **during DR stabilization**	**28.46** ± **12.94 (25–40)**	**42.4** ± **18.3 (30–50)**	**30.8** ± **10.6 (23–35)**	**0.001**	**1.07 (1.03–1.12)**	**1.04 (0.99–1.10)**
**MV (hours)**	**159.96** ± **240.51 (24–228)**	**211.23** ± **273.55 (48–288)**	**46.4** ± **54.2 (9–89)**	**0.02**	**1.02 (1.00–1.03)**	**1.02 (1.01–1.04)**
**Supplemental oxygen (hours)**	**670.44** ± **678.61 (96–1056)**	**1085.51** ± **685.46 (600–1512)**	**258.84** ± **327.65 (30–372)**	**<0.001**	**1.00 (1.002–1.005)**	**1.00 (1.00–1.00)**
*N* (%)						
OIT in the DR	16 **(**15)	7 **(**16.3)	9 **(**14.8)	0.83	1.12 **(**0.37–3.29)	0.68 **(**0.15–2.87)
**Surfactant**	**54 (50.5)**	**32 (72.3)**	**22 (34.9)**	**<0.001**	**4.97 (2.19–11.88)**	**2.08 (0.72–6.09)**
**MV**	**41 (38.3)**	**30 (68.2)**	**11 (17.7)**	**<0.001**	**9.94 (4.13–25.68)**	**3.85 (1.28–11.94)**
**MV at 7 DOL**	**10 (9.3)**	**9 (20.5)**	**1 (1.6)**	**<0.001**	**26649610 (0.00)**	**–**
**Postnatal corticosteroids**	**20 (18.7)**	**19 (43.2)**	**1 (1.6)**	**<0.001**	**47.12 (5.98–371.11)**	**6.81 (1.02–135.7)**
**Other outcomes** ${\bar{{{\boldsymbol{X}}}}}$± **SD (IQR)**						
**Duration of hospitalization (days)**	**63.11** ± **75.21 (40.5–74.50)**	**80.53** ± **113.86 (54–87)**	**51.5** ± **14.57 (40–60)**	**0.02**	**1.02 (1.00–1.04)**	**1.01 (0.99–1.03)**
**NICU length of stay (days)**	**30.42** ± **19.38 (16–40.75)**	**40.51** ± **22.41 (28–58)**	**23.28** ± **12.73 (14–31)**	**<0.001**	**1.06 (1.03–1.09)**	**1.03 (0.99–1.07)**
*N* (%)						
**IVH** > **II**	**6 (5.6)**	**6 (13.6)**	**0 (0)**	**0.002**	**74453420**	**–**
PVL	4 **(**3.7)	1 **(**2.3)	3 **(**4.8)	0.53	0.47 **(**0.02–3.77)	0.19 **(**0.00–3.43)
**ROP** > **II**	**5 (4.7)**	**4 (9.1)**	**1 (1.6)**	**0.03**	**7.75 (1.09–155.02)**	**1.01 (0.08–13.01)**
**Ibuprofen for PDA**	**10 (9.3)**	**8 (18.2)**	**2 (3.2)**	**0.01**	**6.78 (1.59–46.58)**	**2.21 (0.39–17.42)**
**NEC (≥ stage 2)**	**9 (8.4)**	**7 (15.9)**	**2 (3.2)**	**0.02**	**5.68 (1.29–39.49)**	**3.66 (0.52–35.92)**
Isolated intestinal perforation	4 **(**3.7)	3 **(**6.8)	1 **(**1.6)	0.15	4.54 **(**0.56–93.44)	7.13 **(**0.54–188.93)
**Nosocomial sepsis**	**37 (34.6)**	**24 (54.5)**	**13 (20.6)**	**<0.001**	**5.59 (2.4–13.71)**	**2.89 (0.23–9.21)**
**Death**	**11 (10.3)**	**11 (100)**	**0 (0)**	**<0.001**	**–**	**–**

^a^Adjusted by gestational age.

Statistically significant results are shown in bold.

*ANS* antenatal steroids, *AOR* adjusted odds ratio, *BPD* bronchopulmonary dysplasia, *DOL* days of life, *DR* delivery room, *IQR* interquartile range, *IVF* in vitro fertilization, *IVH* intraventricular hemorrhage, *MV* mechanical ventilation, *N* number of subjects, *NEC* necrotizing enterocolitis, *NICU* neonatal intensive care unit, *NIMV* non-invasive mechanical ventilation, *OIT* orotracheal intubation, *PDA* patent ductus arteriosus, *PVL* periventricular leukomalacia, *ROP* retinopathy of prematurity, *SD* standard deviation, *SGA* small for gestational age; $\bar{X} ,\, {{{\rm{mean}}}}$.

### Mitochondrial haplogroups

Mitochondrial haplogroups (Table 2) were studied in patients born to Caucasian mothers (*n* = 90). In these patients, haplogroup H was the most frequent, with a prevalence of 41.1%. There were no significant differences in the distribution of haplogroups between patients in the BPD/death group and those who survived without BPD. When haplogroups were clustered according to phylogenetic origin, a comparable distribution was observed for patients with BPD/death and survivors without BPD (see Table 2 for further details).

**Table 2:** Distribution of mitochondrial haplogroups in patients born to Caucasian mothers.

*N* (%)	Total *N* = 90	BPD/death *N* = 38		No BPD/death *N* = 52	*p*-value	OR	aOR^a^
**Haplogroups**							
H	37 (41.1)	17 (44.7)		20 (38.5)	0.55	1.3 (0.55–3.03)	1.28 (0.42–3.95)
J	4 (4.4)	2 (5.3)		2 (3.8)	0.75	1.39 (0.19–10.33)	0.94 (0.05–19.55)
K	6 (6.7)	2 (5.3)		4 (7.7)	0.65	0.67 (0.12–3.84)	1.14 (0.12–11)
T	3 (3.3)	0 (0)		3 (5.8)	0.13	–	–
U	13 (14.4)	6 (15.8)		7 (13.5)	0.76	1.21 (0.37–3.93)	2.28 (0.50–10.36)
OTHERS	23 (25.6)	10 (26.3)		13 (25)	0.89	1.07 (0.41–2.79)	0.84 (0.22–3.29)
V	1 (1.1)	0 (0)		1 (1.9)	0.39	–	–
SHV	3 (3.3)	1 (2.6)		2 (3.8)	0.75	0.68 (0.06–7.74)	0.36 (0.03–4.74)
**Haplogroup clusters**							
SHV/H/V	41 (45.6)	18 (47.4)		23 (44.2)	0.77	1.14 (0.49–2.63)	1.14 (0.38–3.39)
J/T	7 (7.8)	2 (5.3)		5 (9.6)	0.45	0.52 (0.09–2.85)	0.27 (0.03–2.71)
U/K	19 (21.1)	8 (21.1)		11 (21.2)	0.99	0.99 (0.36–2.77)	1.96 (0.51–7.61)
OTHERS	23 (25.6)	10 (26.3)		13 (25)	0.89	1.07 (0.41–2.79)	0.84 (0.22–3.29)
${\bar{{{\boldsymbol{X}}}}}$± ***SD (IQR)***	**Total** ***N*** = **65**	**BPD/death** ***N*** = **30**	**No BPD/death** ***N*** = **35**		***p***-**value**	**OR**	**aOR**
ccf-mtDNA^b^ (U/µl)	7.76 ± 12.49	8.74 ± 16.44		6.92 ± 7.87	0.56	1 (1–1)	1 (1–1)

^a^Adjusted by gestational age.

^b^ccf-mtDNA levels are expressed every 10,000 U/µl.

*aOR* adjusted odds ratio, *BPD* bronchopulmonary dysplasia, *Ccf-mtDNA* circulating cell-free mitochondrial DNA, *IQR* interquartile range, *mtDNA* mitochondrial DNA, *N* number of patients, *OR* odds ratio, *SD* standard deviation; $X,\, {{{\rm{mean}}}}.$

No significant differences between haplogroups in secondary outcomes were observed, except for a significantly higher prevalence of haplogroup H among patients with moderate-to-severe BPD (*n* = 6 [16.2%] vs. *n* = 2 [3.8%]; *p* = 0.04) and in patients who required intubation in the delivery room (*n* = 9 [24.3%] vs. *n* = 4 [7.5%]; *p* = 0.03) (Supplementary Table S1).

### Circulating cell-free mitochondrial DNA

In our sample, the amount of ccf-mtDNA in the entire population was 77,603 ± 124,974 U/µl and was higher in patients with BPD/death (87,443 ± 164,365 U/µl vs 69,168 ± 78,727 U/µl; *p* = 0.56), although this difference is not significant (Table 2). The only outcome for which an association with ccf-mtDNA levels was observed was the need for intubation in the delivery room, which was associated with higher ccf-mtDNA levels (Table 3).

**Table 3:** Correlation between the CCF-MtDNA levels and variables of interest.

Qualitative variables		*N*	$\bar{X}$± SD	*p*-value
BPD	No	52	64,752 ± 67,401	0.64
	Yes	28	92,968 ± 169,654	
Exitus	No	72	76,939 ± 119,431	0.97
	Yes	8	53,825 ± 31,017	
O2 in the DR	No	6	93,616 ± 7,833	0.22
	Yes	74	73,088 ± 116,922	
**OIT**	**No**	**65**	**69,299** ± **62,995**	**0.007**
	**Yes**	**12**	**105,925** ± **260,866**	
Surfactant	No	35	63,195 ± 51,568	0.86
	Yes	45	83,522 ± 144,973	
GA < 28 weeks	No	50	70,590 ± 69,909	0.69
	Yes	30	81,358 ± 164,175	
Respiratory support 7th DOL	None		62,101 ± 57,261	0.7
	CN		49,875 ± 25,230	
	NIMV		90,640 ± 158,146	
	MV		67,342 ± 39,944	
	HFO		106,000	
Haplogroup clusters	SHV/H/V		86,620 ± 165,662	0.58
	J/T		80,600 ± 45,768	
	U/K		74,628 ± 95,359	
	Others		59,413 ± 45,526	
**Quantitative variables**		***N*** = ***80***	***Rho Spearman*** **(CI 95%)**	***p-value***
GA (weeks)			0.007 (−-0.22-0.23)	0.95
Maximum FiO_2_ during DR stabilization			0.194 (−0.04–0.41)	0.09
FiO_2_ the first 24 hours			0.069 (−0.16-0.29)	0.54
FiO2 the 7th DOL			0.026 (−0.21–0.25)	0.83
Hours on MV			0.099 (−0.25–0.42)	0.57
Supplemental O_2_ (hours)			0.106 (−0.15–0.35)	0.40
NICU length of stay (days)			0.094 (−0,14–0.32)	0.42
Duration of hospitalization (days)			−0.01 (−0.24–0.22)	0.93
Weight at discharge			−0.099 (−0.33–0.15)	0.42
Weight Z-score at discharge			−0.06 (−0.31–0.19)	0.63

Statistically significant results are shown in bold.

*BPD* bronchopulmonary dysplasia, *ccf-mtDNA* circulating cell-free mitochondrial DNA, *DR* delivery room, *DOL* days of life, *FiO*_*2*_ fraction of inspired oxygen, *GA* gestational age, *MV* mechanical ventilation, *N* number of patients, *NICU* neonatal intensive care unit, *O*_*2*_ Oxygen, *OIT* orotracheal intubation, *SD* standard deviation; $\bar{X} ,\, {{{\rm{mean}}}}.$

**Fig. 2:**
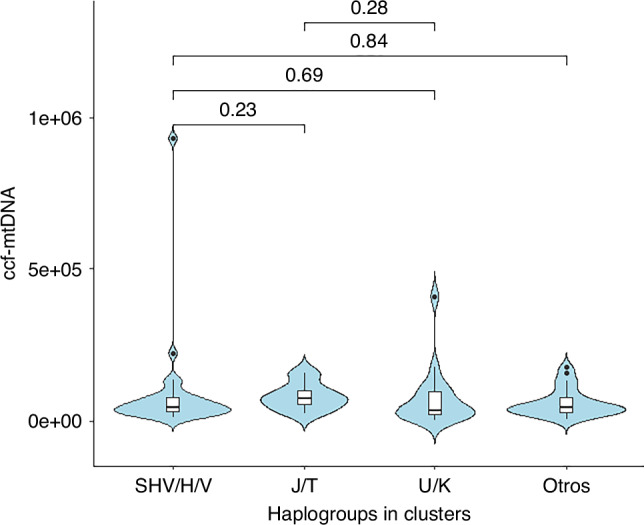
Violin plot showing the distribution of ccf-mtDNA levels (U/μL) across different haplogroups (SHV/H/V, J/T, U/K, Others). Values above the lines reflect the p-values for the corresponding statistical comparisons.

Ccf-mtDNA levels did not differ between haplogroups (Fig. 2). When we stratified by cases and controls, we observed no association between ccf-mtDNA levels and haplogroups (Supplementary Table S2).

No significant differences have been found in the results regarding mtDNA in patients born to Caucasian and non-Caucasian mothers (Supplementary Table S3).

## Discussion

In this prospective cohort study, we explored the correlation between the distribution of mtDNA haplogroups and ccf-mtDNA levels at birth and the combined outcome of BPD or death in very preterm infants admitted to NICU. We observed no significant differences between these mitochondria-related variables and either BPD/death or other secondary respiratory outcomes. Therefore, our findings do not support a significant role of mtDNA haplogroups in the development of BPD.

While mitochondrial function is known to be important in the neonatal lung,^9^ there is limited information on its role in BPD. Although BPD pathogenesis is complex and multifactorial, evidence suggests that OS and inflammation play key roles.^26,27^ At birth, premature infants with respiratory distress syndrome due to lung immaturity usually require prolonged respiratory support^6^ and higher oxygen concentrations. These conditions increase ROS production, contributing to BPD development,^9^ and are also linked to mitochondrial dysfunction and mtDNA mutations.^6^

Predicting which newborns will develop BPD remains a challenge. Numerous models based on clinical variables^4^ have been developed to identify high-risk patients and individualize care, and to aid the development of more effective preventive strategies. However, to date none have been fully incorporated into clinical practice.

Considering the limitations of current predictive models, identifying new biomarkers, such as mtDNA polymorphisms, could enhance our ability to predict BPD susceptibility.

Certain mtDNA polymorphisms are known as mtDNA haplogroups.^28^ Previous studies on mtDNA variation have demonstrated links between these haplogroups and alterations in mitochondrial function, including the modified production of ROS and mtDNA damage,^8,9^ as well as increased individual susceptibility to certain diseases.^29^ In 2012, Zheng et al. ^30^ investigated the link between mitochondrial DNA haplogroups and susceptibility to chronic obstructive pulmonary disease, and they found that mtDNA haplogroups A and M7 can increase the risk of developing this condition, while haplogroups D, F, and M9 may act as protective factors. Some years later, Farha et al. ^31^ identified an association between haplogroup M and clusters UK, HV, and JT with an increased risk of pulmonary hypertension compared to haplogroup L (L0/1/2 and L3). Certain European haplogroups, like haplogroup U, are associated with higher total serum IgE levels and may increase the risk of atopy-related conditions.^32^ Therefore, we hypothesized that certain mtDNA haplogroups may increase individual predisposition to BPD. To our knowledge, this is the first study to investigate the hypothesis that mitochondrial haplogroups influence the risk of developing BPD.

Haplogroups have also been studied as potential risk predictors in other diseases.^33–36^ Haplogroup H, which is the most prevalent in Europe,^37^ may increase the risk for some cardiovascular diseases such as idiopathic dilated cardiomyopathy,^33^ hypertrophic cardiomyopathy,^37^ and ischemic cardiomyopathy.^36^ On the other hand, some studies have shown that haplogroup J acts as a protective factor for ischemic cardiomyopathy,^36^ osteoarthritis^38^ and cognitive decline in Parkinson disease.^35^ In our study population, haplogroup H was the most common (41.1%), and was significantly more prevalent in patients with moderate-to-severe BPD (*n* = 6 [75%] vs. *n* = 2 [25%]; *p* = 0.04), although haplogroup H does not appear to increase the overall risk of developing the combination of BPD/death.

Jeong et al. ^13^ used next-generation sequencing to analyze somatic mtDNA mutations caused by oxygen exposure in 10 extremely preterm infants. They identified certain mtDNA mutations present at birth that can impair energy production and contribute to lung inflammation.^13^ The authors observed that the number of mutations increased in the first weeks due to rising ROS levels but later decreased, suggesting that changes in clinical management could potentially reverse mtDNA damage.

When mitochondria are damaged due to an increase in reactive oxygen species that saturate antioxidant mechanisms,^9,39^ ccf-mtDNA is released into the cytosol, and its levels increase in parallel with those of pro-inflammatory cytokines, such as tumor necrosis factor alpha and interleukin 6.^40^ These molecules can act as DAMPs, triggering immune and inflammatory responses.^14^ Ccf-mtDNA levels are not stable and can change considerably in a short time depending on stressors, mean arterial pressure, white blood cell count, and other factors.^40^ They are increased in some infectious conditions (COVID19,^14^ sepsis,^40^ and acute respiratory distress syndrome^16^) and in response to major surgery and trauma.^18^ Moreover, ccf-mtDNA levels are correlated with increased severity and mortality rates in hospitalized critically ill patients^17^ and in patients with acute lung injury.^15^

Although biologically plausible, little is known about associations between BPD and other respiratory conditions. Elevated levels of ccf-mtDNA are linked to a higher annual rate of COPD exacerbations due to their pro-inflammatory characteristics.^41^ Furthermore, patients with asthma have higher ccf-mtDNA levels compared with healthy controls,^42^ although levels are not related to asthma severity or control.^42^

In our cohort, patients who developed BPD/death had higher levels of ccf-mtDNA in the first 24 hours of life compared with those who survived without BPD, although this difference was not significant. This difference could be explained by variation in ccf-mtDNA levels due to increases or decreases in platelet levels, which can result in increases in vesicles containing mtDNA that is then released into the plasma.^40^ We observed a correlation between ccf-mtDNA copies and intubation in the delivery room. This is likely because patients who require intubation are born in a more critical condition. However, this observation should be interpreted with caution, as it is based on a small sample of patients (*n* = 12) with some extreme determinations, therefore, it is not enough to reach conclusions. We observed no other correlations between ccf-mtDNA levels and respiratory or outcome-related variables.

Our study is the first to investigate mitochondrial haplogroups and ccf-mtDNA levels in very preterm infants. Another key strength is that the study had a relatively large sample size for a “discovery” cohort, which was intended to identify potential biomarkers of BPD. Several limitations of the study should be noted. First, it is a single-center study, although uniform perinatal management may be considered positive. Second, in our analysis and comparison of haplogroups we excluded patients born to non-Caucasian mothers, in order to minimize potential biases. Nonetheless, a total of 23 patients fell into the “other haplogroups” group, which probably includes non-European haplogroups. Third, the lack of long-term respiratory outcomes is also a limitation, as these data could help future studies better understand the lasting impact of mitochondrial dysfunction in preterm infants. Finally, our findings did not support our study hypothesis, which is always a disappointing result of an investigation. However, we strongly believe that the publication and dissemination of positive, negative, and neutral results is essential for the progress of science.

In conclusion, we observed no significant association between common European haplogroups and ccf-mtDNA levels in the first 24 hours of life and the risk of BPD/death. Further studies with larger patient populations from different continents are necessary to determine whether any mitochondrial-related variables predispose individuals to the development of BPD. This could help us identify patients at greater risk of developing BPD early, allowing us to implement measures promptly and potentially improve the course of the disease.

## Supplementary information

Supplementary Table S3

Supplementary Table S1

Supplementary Table S2

## Data Availability

The data used in this study are available upon reasonable request from the corresponding author.
