# Supplementary material for: Mitochondrial DNA haplogroups and circulating cell-free mitochondrial DNA as biomarkers of bronchopulmonary dysplasia
Source: Pediatr Res. 2025 Apr 17;98(6):2292–9. doi: 10.1038/s41390-025-04052-7 (PMC12811138; doi:10.1038/s41390-025-04052-7)
Supplement: Supplementary file 1 — Supplementary Table S3 [file 41390_2025_4052_MOESM1_ESM.pdf]

Supplemental Table S3. Results regarding mtDNA in patients born out of Caucasian mothers and non-Caucasian mothers

N (%)	*Total n=107*	*BPD/death n=43*	*No BPD/death n=64*	*p-value*	*OR*	*aOR*[a]
**Haplogroups**						
H	38 (35.5)	18 (41.9)	20 (31.3)	0.27	1.3 (0.56-2.81)	1.57 (0.55-4.53)
J	5 (4.7)	3 (7)	2 (3.1)	0.35	1.85 (0.28-15.31)	1.17 (0.07-19.23)
K	7 (6.5)	2 (4.7)	5 (7.8)	0.37	0.49 (0.07-2.62)	0.66 (0.08-5.22)
T	4 (3.7)	1 (2.3)	3 (4.7)	0.44	0.41 (0.02-3.55)	0.81 (0.05-12.53)
U	14 (13.1)	6 (14)	8 (12.5)	0.58	0.93 (0.26-3.19)	2.11 (0.50-8.99)
OTHERS	35 (32.7)	14 (32.6)	21 (32.8)	0.47	0.89 (0.35-2.21)	0.54 (0.16-1.75)
V	1 (0.93)	0 (0)	1 (1.6)	0.58	-	-
SHV	3 (2.8)	1 (2.3)	2 (3.1)	0.62	0.62 (0.03-6.99)	0.35 (0.03-4.65)
**Haplogroup clusters**						
SHV/H/V	42 (39.3)	19 (44.2)	23 (35.9)	0.37	0.61 (-0.57-0.99)	1.27 (0.45-3.57)
J/T	9 (8.4)	4 (9.3)	5 (7.8)	0.57	1.07 (0.24-4.6)	0.96 (0.14-6.86)
U/K	21 (19.6)	8 (18.6)	13 (20.3)	0.44	0.82 (0.27-2.38)	1.48 (0.43-5.17)
OTHERS	35 (32.7)	14 (32.6)	21 (32.8)	0.47	0.89 (0.35-2.21)	0.54 (0.16-1.75)
$\bar{X}$± SD (IQR)	***Total n=80***	***BPD/death n=37***	***No BPD/death n=43***	***p-value***	***OR***	***aOR***
Ccf-mtDNA[b] (U/µl)	7.46 ± 11.38	8.76 ± 15.22	6.45 ± 7.15	0.49	1 (1-1)	1 (1-1)

a Adjusted by gestational age

b Ccf-mtDNA levels are expressed every 10,000 U/µl

aOR, adjusted odds ratio; BPD, bronchopulmonary dysplasia; Ccf-mtDNA, circulating cell-free mitochondrial DNA; IQR, interquartile range; mtDNA, mitochondrial DNA; N, number of patients; OR, odds ratio; SD, standard deviation; X, mean.
