# Supplementary material for: Mitochondrial DNA haplogroups and circulating cell-free mitochondrial DNA as biomarkers of bronchopulmonary dysplasia
Source: Pediatr Res. 2025 Apr 17;98(6):2292–9. doi: 10.1038/s41390-025-04052-7 (PMC12811138; doi:10.1038/s41390-025-04052-7)
Supplement: Supplementary file 2 — Supplementary Table S1 [file 41390_2025_4052_MOESM2_ESM.pdf]

N (%)		*Haplogroup H N=37*	*Haplogroup other than H N=53*	*p-value*	*Cluster SHV/H/V N=41*	*Cluster other than SHV/H/V N=49*	*p-value*
BPD	Yes	14 (37.8)	15 (28.3)	0.34	15 (36.6)	14 (28.6)	0.42
	No	23 (62.2)	38 (71.7)		26 (63.4)	35 (71.4)	
BPD moderate-to-severe	**Yes**	**6 (16.2)**	**2 (3.8)**	**0.04**	6 (14.6)	2 (4.1)	0.08
	**No**	**31 (83.8)**	**51 (96.2)**		35 (85.4)	47 (95.9)	
Supplemental O_2 in the DR	Yes	36 (97.3)	50 (94.3)	0.50	40 (97.6)	46 (93.9)	0.39
	No	1 (2.7)	3 (5.7)		1 (2.4)	3 (6.1)	
OIT in the DR	**Yes**	**9 (24.3)**	**4 (7.5)**	**0.03**	9 (21.9)	4 (8.2)	0.06
	**No**	**28 (75.7)**	**49 (92.5)**		32 (78)	45 (91.8)	
MV at 7 DOL	Yes	4 (10.8)	5 (9.4)	0.85	5 (12.2)	4 (8.2)	0.56
	No	30 (81.1)	43 (81.1)		33 (80.5)	40 (81.6)	
Death	Yes	4 (10.8)	5 (9.4)	0.83	4 (9.8)	5 (10.2)	0.94
	No	33 (89.2)	48 (90.6)		37 (90.2)	44 (89.8)	
$\overline{X}$ ± SD							
Maximum FiO_2 in the DR		38.9 ± 17.6	34.5 ± 14.9	0.21	37.1 ± 18.1	35.5 ± 14.4	0.65
FiO_2 7th DOL		23.6 ± 5.6	23 ± 5.4	0.61	23.5 ± 5.4	23.1 ± 5.5	0.70
Hours on MV		251.7 ± 379.4	115.3 ± 117.1	0.13	210.3 ± 357.3	133.9 ± 116.4	0.39
Supplemental O_2 (hours)		683.5 ± 730.5	631.1 ± 673.4	0.75	663.4 ± 701.8	642.9 ± 693.6	0.90
NICU length of stay (days)		31.8 ± 20.4	28.6 ± 18.6	0.46	30.9 ± 20.4	29.1 ± 18.5	0.66
Duration of hospitalization (days)		58.8 ± 26.1	69.2 ± 104.1	0.55	58.8 ± 25.2	70.1 ± 108.4	0.52

Supplementary Table S1. Correlation between haplogroups and selected outcomes in patients born to Caucasian mothers.

Statistically significant results are shown in bold.

BPD, bronchopulmonary dysplasia; DOL, days of life; DR, delivery room; FiO_2, fraction of inspired oxygen; MV, mechanical ventilation; NICU, neonatal intensive care unit; O_2, oxygen; OIT, orotracheal intubation; SD, standard deviation.
