# Supplementary material for: Mitochondrial DNA haplogroups and circulating cell-free mitochondrial DNA as biomarkers of bronchopulmonary dysplasia
Source: Pediatr Res. 2025 Apr 17;98(6):2292–9. doi: 10.1038/s41390-025-04052-7 (PMC12811138; doi:10.1038/s41390-025-04052-7)
Supplement: Supplementary file 3 — Supplementary Table S2 [file 41390_2025_4052_MOESM3_ESM.pdf]

Patients with the combined variable BPD/death

Haplogroups		*N*	$\overline{X} \pm SD$	*p-value*
H	No	22	64,445 ± 44,191	0.32
	Yes	15	114,306 ± 228,412	
J	No	34	85,394 ± 154,889	0.92
	Yes	3	76,333 ± 28,976	
K	No	35	87,725 ± 152,180	0.61
	Yes	2	31,000 ± 10,323	
T	No	36	84,991 ± 150,565	0.94
	Yes	1	72,700	
U	No	33	90,187 ± 156,073	0.52
	Yes	4	39,050 ± 39,917	
OTHERS	No	26	88,438 ± 174,884	0.82
	Yes	11	75,727 ± 52,663	
V	No	37	84,659 ± 148,473	-
	Yes	0		
SHV	No	36	85,208 ± 150,541	0.89
	Yes	1	64,900	
Haplogroup clusters				
SHV/H/V	No	21	64,423 ±45,282	0.35
	Yes	16	111,218 ± 221,012	
J/T	No	33	85,778 ± 157,274	0.89
	Yes	4	75,425 ± 23,728	
U/K	No	31	94,006 ± 160,407	0.39
	Yes	6	36,366 ± 31,537	
OTHERS	No	26	88,438 ± 174,884	0.82
	Yes	11	75,727 ± 52,663	

Patients in control group

Haplogroups		*N*	$\bar{X} \pm SD$	*p-value*
H	No	31	69,452 ± 77,384	0.62
	Yes	12	57,066 ± 61,649	
J	No	42	66,950 ± 73,441	0.58
	Yes	1	25,900	
K	No	38	60,280 ± 50,958	0.56
	Yes	5	109,440 ± 170,310	
T	No	42	63,853 ± 72,329	0.22
	Yes	1	156,000	
U	No	36	62,776 ± 76,004	0.52
	Yes	7	82,557 ± 55,237	
OTHERS	No	28	75,932 ± 85,169	0.14
	Yes	15	47,449 ± 36,845	
V	No	43	65,996 ± 72,831	-
	Yes	0		
SHV	No	41	65,940 ± 74,553	0.98
	Yes	2	67,150 ± 21,283	
Haplogroup clusters				
SHV/H/V	No	29	69,611 ± 79,996	0.65
	Yes	14	58,507 ± 57,133	
J/T	No	41	64,779 ± 72,975	0.63
	Yes	2	90,950 ± 91,994	
U/K	No	31	55,249 ± 49,502	0.27
	Yes	12	93,758 ± 111,372	
OTHERS	No	28	75,932 ± 85,169	0.14
	Yes	15	47,449 ± 36,845	

Supplementary Table S2. Analysis of the association between haplogroups and ccf-mtDNA levels, stratified by cases and controls.

BPD, bronchopulmonary dysplasia; ccf-mtDNA, circulating cell-free mitochondrial DNA; N, number of patients; SD, standard deviation.
